# Primary care consultation rates among people with and without severe mental illness: a UK cohort study using the Clinical Practice Research Datalink

**DOI:** 10.1136/bmjopen-2015-008650

**Published:** 2015-12-16

**Authors:** Evangelos Kontopantelis, Ivan Olier, Claire Planner, David Reeves, Darren M Ashcroft, Linda Gask, Tim Doran, Siobhan Reilly

**Affiliations:** 1 Centre for Health Informatics, Institute of Population Health, University of Manchester, Manchester, UK; 2 NIHR School for Primary Care Research, Centre for Primary Care, Institute of Population Health, University of Manchester, Manchester, UK; 3 Manchester Institute of Biotechnology, University of Manchester, Manchester, UK; 4 Centre for Biostatistics, Institute of Population Health, University of Manchester, Manchester, UK; 5 Centre for Pharmacoepidemiology and Drug Safety, Manchester Pharmacy School, University of Manchester, Manchester, UK; 6 Department of Health Sciences, University of York, York, UK; 7 Division of Health Research, University of Lancaster, Lancaster, UK

**Keywords:** MENTAL HEALTH, PRIMARY CARE, PUBLIC HEALTH, PSYCHIATRY

## Abstract

**Objectives:**

Little is known about service utilisation by patients with severe mental illness (SMI) in UK primary care. We examined their consultation rate patterns and whether they were impacted by the introduction of the Quality and Outcomes Framework (QOF), in 2004.

**Design:**

Retrospective cohort study using individual patient data collected from 2000 to 2012.

**Setting:**

627 general practices contributing to the Clinical Practice Research Datalink, a large UK primary care database.

**Participants:**

SMI cases (346 551) matched to 5 individuals without SMI (1 732 755) on age, gender and general practice.

**Outcome measures:**

Consultation rates were calculated for both groups, across 3 types: face-to-face (primary outcome), telephone and other (not only consultations but including administrative tasks). Poisson regression analyses were used to identify predictors of consultation rates and calculate adjusted consultation rates. Interrupted time-series analysis was used to quantify the effect of the QOF.

**Results:**

Over the study period, face-to-face consultations in primary care remained relatively stable in the matched control group (between 4.5 and 4.9 per annum) but increased for people with SMI (8.8–10.9). Women and older patients consulted more frequently in the SMI and the matched control groups, across all 3 consultation types. Following the introduction of the QOF, there was an increase in the annual trend of face-to-face consultation for people with SMI (average increase of 0.19 consultations per patient per year, 95% CI 0.02 to 0.36), which was not observed for the control group (estimates across groups statistically different, p=0.022).

**Conclusions:**

The introduction of the QOF was associated with increases in the frequency of monitoring and in the average number of reported comorbidities for patients with SMI. This suggests that the QOF scheme successfully incentivised practices to improve their monitoring of the mental and physical health of this group of patients.

**Strengths and limitations of this study**

- Currently the largest longitudinal study of consultation rate patterns for people with severe mental illness (SMI) in UK primary care, covering 2000–2012.
- In total, 346 551 individuals with SMI matched to five individuals without SMI on age, gender and general practice.
- Provides evidence on the increase of face-to-face consultation rates for people with SMI, compared with controls, potentially driven by the Quality and Outcomes Framework incentivisation scheme.
- However, we could not assess the context of the consultations and do not know whether they adequately addressed patients’ needs.

## Introduction

Primary care is well positioned to provide timely and accessible healthcare services to people with severe mental illness (SMI).^1^ Between 2009 and 2010, general practices in the UK were the only known healthcare provider for approximately 31% of people on practice SMI registers,^2^ and this figure could rise under new National Health Service (NHS) arrangements, with secondary care mental health services under increasing pressure to discharge people back to primary care earlier.^3^ ^4^

It is therefore important to monitor aspects of service utilisation, and primary care consultation rates are one such measure, vital for commissioning and resource planning.^5^ Consultation rates can be indicative of the level of need of a specific patient group and also identify inequalities in the provision of key primary care services for that group, when compared with the general population.^6^ Previous research has examined whether the consultation rate patterns of people with mental illness who die by suicide differ to the patterns observed for the general population,^7^ but to date, there has been no investigation on the consultation patterns of people with SMI and how they compare to those of the general population.

The consultation is the main setting through which primary care services are delivered, including diagnosis, treatment, monitoring, health promotion and preventative activities.^8^ However, in the UK, primary care activities have been found to be suboptimal for these patients. People with SMI are less likely to receive cardiovascular disease (CVD) screening, compared with people with diabetes,^9^ and CVD checks are not always performed in accordance with national guidance.^10^

This raises concerns because the physical health of people with SMI is much poorer, compared with the general population. People with SMI are at greater risk of developing serious long-term physical health conditions when compared with people without,^11^ ^12^ and are more likely to die prematurely.^13^ Furthermore, the main cause of death in this population is CVD; a preventable condition routinely managed in primary care.^11^ Primary care is an appropriate setting for managing people with SMI and has a workforce ideally suited to deliver care to this population.^14^ However, improvements to modern primary healthcare appear not to benefit this vulnerable population, as evidenced by a widening morbidity and mortality gap.^15^ This could be attributed to the fact that health professionals need additional training to be able to care for people with SMI effectively.^16^ ^17^

In the UK, national data on primary care consultations are not routinely collected.^5^ We are aware of only one study which examined consultation patterns over time, from 1995 to 2009.^18^ The study defined consultations as ‘direct contact between a clinician and patient’ and found that rates in the general population increased over time. On average, a patient had 3.9 consultations per year in 1995/1996, rising to 5.4 in 2008/2009. However, consultation patterns of patient subpopulations, and specifically of people with SMI, were not investigated.

Little is known about service utilisation rates for people with SMI. Research carried out in the UK nearly two decades ago found that people with schizophrenia were more likely to consult general practitioners (GPs) than other primary care staff but did not report primary care consultation rates.^19^ Recently, rates have been reported to be as low as 4.3 per year,^2^ a figure only slightly higher than that reported for the general population.^18^ However, the characteristics of people with SMI are different to those of the general population and consultation levels might be very different between the two groups when adjusted for known predictors of service utilisation.

Clarity on consultation patterns would also shed light on the effect of the introduction of the Quality and Outcomes Framework (QOF) in 2004 on primary care service utilisation. The QOF is a national financial incentive scheme that rewards general practices for the treatment and management of chronic conditions.^20^ Management of people with SMI has been incentivised since the start of the scheme, although there have been several changes to the quality indicators over time. In 2011/2012, the quality indicators for all patients with SMI included alcohol consumption screening; body mass index, blood glucose, blood pressure and total cholesterol monitoring; and care plan documentation. Additional indicators for subgroups included cervical screening for females, and lithium and serum creatinine level monitoring for those prescribed lithium. Nationally, incentive payments to practices for managing patients with SMI total £40 million per year.

We attempt to address the knowledge gap with this longitudinal observational study, which investigates consultation rates in people with SMI and in a control group without SMI, matched on age, sex and general practice. We used routinely collected data from clinical computer systems and uploaded to a large primary care database, the Clinical Practice Research Datalink (CPRD). More specifically, our aims were to: (1) report the recorded primary care consultation patterns by type (face-to-face, telephone and other) for people with an SMI diagnosis and their controls, over the study period; (2) investigate the associations between age, gender, SMI diagnosis and other comorbidities with primary care consultations; (3) compare primary care consultation frequency between people with SMI and controls, after controlling for other factors; and (4) assess the effect of the introduction of the QOF on consultation frequency for both people with SMI and controls. A detailed investigation of comorbidity patterns and their changes over time has been published elsewhere.^21^

## Methods

### The database

The CPRD is a large computerised database of anonymised primary care medical records. It contains complete patient information for participating practices, with the healthcare events (diagnoses, treatments, referrals, tests and prescriptions) recorded using coding systems (Read coding for diagnoses). Practice characteristics are described in detail elsewhere.^22^ The database is broadly representative of the UK population, although larger practices are over-represented.

Practices need to meet prespecified data entry quality criteria to be defined as ‘up to research standard’, and for each study year, our main sample included all CPRD practices that were classed as such for the whole year. We also generated two data sets to test the sensitivity of our findings. First, we included all practices contributing data across the entire study period. Second, we included a subsample of 50 practices, representative of UK practices in terms of area deprivation,^23^ and practice list size.^24^

### Defining people with SMI and controls

Information was extracted for the period 1 April 2000 to 31 March 2012 and aggregated into 12 yearly ‘bins’, to correspond with financial years 2000/2001–2011/2012.

We used Read codes to identify the presence of SMI. First, we identified relevant keywords (or key-stubs) and codes, for example ‘paranoi’ and ‘E100.00’ (simple schizophrenia). Next, the CPRD was searched for codes that matched the list in either the code or the description field. Finally, the matched code list was reviewed by clinical experts and a final conservative list of codes was agreed.^25^ A similar process was used to define comorbidities (hypertension, asthma, hypothyroidism, osteoarthritis, chronic kidney disease, coronary heart disease, epilepsy, chronic obstructive pulmonary disease, cancer, stroke, heart failure, rheumatoid arthritis, dementia and psoriasis). All code lists we used are available from http://www.clinicalcodes.org.^26^ All conditions, bar asthma, were treated as unresolvable (ie, permanent).

Within each year, all patients registered with a CPRD practice for the whole year and aged 18 or over were eligible for inclusion. The final SMI Read code list was used to identify cases of SMI, which were then grouped into three broad subcategories, in line with the diagnoses used when compiling primary care QOF SMI registers^27^: schizophrenia; affective psychoses (bipolar disorder or other unspecified affective psychosis); other types of psychosis. In the event that an individual received more than one SMI diagnosis over the study period, we used the last available diagnosis to retrospectively ‘correct’ the original diagnosis (ie, we assumed that the latest diagnosis was the correct one). Within each year, each SMI case was then matched on age, sex and practice to five randomly selected patients not associated with SMI up until that time point. More details on the extraction of the cohort have been provided elsewhere,^21^ and a flow chart of the data extraction process is available in the online appendix figure A2.

### Defining consultation type

We defined a ‘consultation’ as involving direct contact between a patient and a healthcare professional within the primary care setting. We divided consultations into two main categories: face-to-face (our primary outcome), and by telephone (see online appendix table A1). We also constructed a third ‘other’ grouping of all other activities that are captured by the ‘consultation type’ codes within the CPRD. This includes mail/email contact, third party consultations (including referrals), secondary care episodes, other administrative tasks and consultations of unknown content. This group is highly heterogeneous and includes many activities that cannot be classed as consultations. However, we decided to use this grouping as an aggregate secondary outcome since it can potentially provide insight into the overall workload associated with patient care in the primary care context. We decided against breaking down the ‘other’ group in more subcategories as we are very doubtful regarding the reliability and across practice consistency of the coding within these ‘other’ categories. In instances where a patient had two or more consultations within a day, we conservatively assumed a single consultation took place, to reduce the likelihood of including duplicate records.^27^ ^29^

### Analyses

Analyses were performed using Stata V.13.1 with an α level of 5%. Aggregate unadjusted consultation rates were calculated for each year and patient group, as the total number of consultations over the number of patients. Multilevel multiple Poisson regression analyses assessed the relationships between consultation rates and patient and practice characteristics, across the three data sets (main and sensitivity 1 and 2). We used the *xtpoisson* command with observations clustered within patients. SMI and control groups were analysed in separate models, to identify potential predictors of: year (as a categorical variable, from 2000/2001 to 2011/2012), consultation type (face-to-face, telephone, other), gender, age (continuous in years) and all the comorbidities previously mentioned as binary. A third model was used to generate adjusted consultation levels and included both groups and the respective covariate (group), as well as interaction terms for group-year, group-consultation type, group-gender, group-age, year-consultation type and group-year-consultation type. A simple interrupted time-series analysis^30^ was conducted using the *itsa* command^31^ to quantify the effect of the QOF on level and trend changes within each group and each consultation type. We included all time points with the exception of 2003/2004 from the analysis, in line with previous approaches,^32^ ^33^ since information on the QOF was in the public domain that year and practices were preparing for the implementation of the scheme in 2004/2005. Since linearity is a vital interrupted time-series assumption, we tested all investigated trends for linearity. Estimates across SMI and control models were compared using the z-score formula. Data were complete and hence approaches to deal with missing data were not required.

## Results

The number of practices included in the analysis varied over the 12-year study period, with 434 practices in 2000/2001, increasing to 569 in 2006/2007 and reducing to 499 in 2011/2012. Prevalence of SMI increased over time from 0.52% in 2000/2001 to 0.63% in 2011/2012, with a greater increase in practices in the most deprived areas (from 0.60% in 2000/2001 to 0.79% in 2011/2012). Mean age was relatively constant over the time period (≈51) but SMI appeared to be diagnosed earlier as time went on, with mean years with the condition increasing from 11.7 in 2000/2001 to 13.2 in 2011/2012. The percentage of people with SMI that were male also increased over the study period, from 45.4% to 48.9%. The mean number of comorbidities was similar for SMI and control cases in 2000/2001 (0.6 and 0.5, respectively), but by 2011/2012 this had increased to 1.0 for people with SMI compared with 0.6 for controls (table 1). The final available diagnosis in terms of subgroup categorisation agreed with the first diagnosis for 75.9% of cases, within a week or less. Agreement within a year and within 5 years increased to 80.1% and 87.2%, respectively.

**Table 1:** Characteristics for severe mental illness (SMI) and control cases, over time

	2000/2001	2001/2002	2002/2003	2003/2004	2004/2005	2005/2006	2006/2007	2007/2008	2008/2009	2009/2010	2010/2011	2011/2012
SMI prevalence												
Overall	0.52	0.52	0.55	0.56	0.57	0.58	0.59	0.60	0.60	0.61	0.62	0.63
By deprivation quintile*												
0 (least deprived)	0.44	0.44	0.45	0.46	0.47	0.48	0.48	0.49	0.48	0.49	0.49	0.50
1	0.46	0.47	0.50	0.51	0.53	0.54	0.54	0.55	0.56	0.56	0.57	0.58
2	0.51	0.52	0.53	0.53	0.54	0.54	0.55	0.56	0.57	0.58	0.58	0.58
3	0.55	0.56	0.58	0.61	0.63	0.64	0.65	0.66	0.66	0.68	0.69	0.70
4	0.60	0.61	0.65	0.67	0.68	0.70	0.72	0.75	0.74	0.75	0.77	0.79
By English region												
North East	0.49	0.51	0.53	0.53	0.54	0.55	0.57	0.64	0.62	0.64	0.67	0.69
North West	0.54	0.56	0.60	0.62	0.65	0.67	0.67	0.68	0.68	0.69	0.72	0.74
Yorkshire and Humber	0.67	0.60	0.59	0.60	0.60	0.59	0.59	0.60	0.65	0.68	0.67	0.75
East Midlands	0.45	0.46	0.49	0.51	0.54	0.54	0.56	0.53	0.53	0.57	0.57	0.65
West Midlands	0.46	0.47	0.49	0.50	0.50	0.50	0.51	0.52	0.51	0.51	0.52	0.53
East England	0.50	0.52	0.53	0.53	0.54	0.54	0.55	0.57	0.58	0.58	0.61	0.63
South West	0.55	0.54	0.54	0.53	0.52	0.51	0.51	0.49	0.50	0.52	0.52	0.52
South Central	0.45	0.45	0.47	0.48	0.49	0.50	0.50	0.52	0.51	0.51	0.51	0.51
London	0.58	0.60	0.64	0.65	0.66	0.68	0.69	0.71	0.71	0.73	0.73	0.73
South East	0.40	0.41	0.43	0.45	0.48	0.50	0.51	0.52	0.54	0.54	0.55	0.55
By country												
England	0.51	0.51	0.53	0.54	0.55	0.56	0.57	0.58	0.58	0.60	0.61	0.63
Northern Ireland	0.48	0.48	0.50	0.53	0.55	0.56	0.58	0.62	0.64	0.64	0.67	0.69
Scotland	0.73	0.74	0.71	0.68	0.70	0.71	0.71	0.72	0.71	0.72	0.72	0.73
Wales	0.48	0.50	0.54	0.59	0.61	0.62	0.63	0.64	0.64	0.65	0.65	0.65
Characteristics												
Number of practices	434	472	503	532	553	566	569	565	565	556	534	499
Practice population	3 805 086	4 199 071	4 534 974	4 843 511	5 071 047	5 214 673	5 321 351	5 369 370	5 449 547	5 432 224	5 301 520	5 069 748
Number of SMI cases	19 658	22 039	24 740	26 969	29 040	30 286	31 267	32 175	32 666	33 117	32 787	31 807
Number of control cases	98 290	110 195	123 700	134 845	145 200	151 430	156 335	160 875	163 330	165 585	163 935	159 035
Mean (SD) age†												
SMI cases	51.6 (17.6)	51.4 (17.5)	51.2 (17.5)	50.9 (17.4)	50.8 (17.2)	50.8 (17.1)	50.8 (17.0)	50.9 (16.9)	51.1 (16.8)	51.2 (16.8)	51.4 (16.8)	51.6 (16.7)
Control cases	51.6 (17.6)	51.4 (17.5)	51.2 (17.5)	50.9 (17.4)	50.8 (17.2)	50.8 (17.1)	50.8 (17.0)	50.9 (16.9)	51.1 (16.8)	51.2 (16.8)	51.4 (16.8)	51.6 (16.7)
Mean (SD) years with SMI	11.7 (11.5)	11.8 (11.6)	11.8 (11.6)	11.8 (11.6)	11.8 (11.6)	12.1 (11.7)	12.2 (11.7)	12.3 (11.6)	12.6 (11.7)	12.8 (11.7)	13.0 (11.7)	13.2 (11.8)
Percentage male†												
SMI cases	45.42	46.18	47.10	47.75	48.02	48.42	48.49	48.89	48.63	48.86	48.82	48.93
Control cases	45.42	46.18	47.10	47.75	48.02	48.42	48.49	48.89	48.63	48.86	48.82	48.93
Mean (SD) no. comorbidities‡												
SMI cases	0.6 (1.0)	0.7 (1.0)	0.7 (1.0)	0.7 (1.1)	0.7 (1.1)	0.8 (1.1)	0.8 (1.2)	0.9 (1.2)	0.9 (1.3)	0.9 (1.3)	0.9 (1.3)	1.0 (1.3)
Control cases	0.5 (0.9)	0.6 (0.9)	0.6 (1.0)	0.6 (1.0)	0.6 (1.0)	0.6 (1.0)	0.6 (1.1)	0.6 (1.1)	0.6 (1.1)	0.6 (1.1)	0.6 (1.1)	0.6 (1.1)

*As measured by the 2007 Index of Multiple Deprivation (IMD), a composite score across seven domains: income, employment, health deprivation and disability, education and skills, barriers to housing, crime and living environment.^23^

†Identical across the two groups since we matched on age, sex and practice.

‡From the preselected group of conditions including hypertension, diabetes, asthma, hypothyroidism, osteoarthritis, chronic kidney disease, coronary heart disease, epilepsy, chronic obstructive pulmonary disease, cancer, stroke, heart failure, rheumatoid arthritis, dementia and psoriasis.

**Figure 1:**
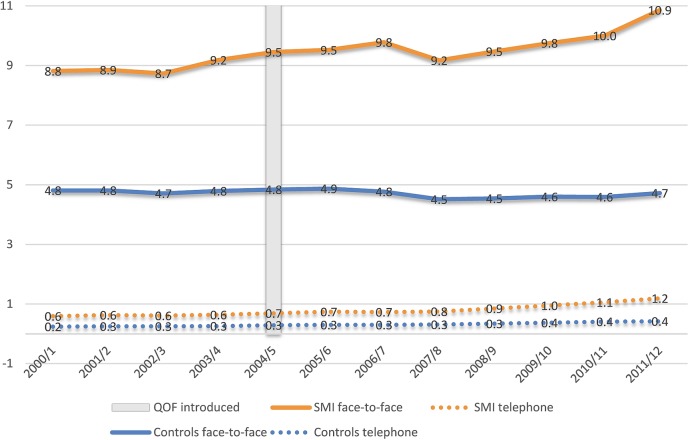
Face-to-face and telephone consultation rates over time for SMI and controls (SMI, severe mental illness; QOF, Quality and Outcomes Framework).

Overall consultation rates increased for both SMI and control cases over time (figure 1). Between 2000/2001 and 2011/2012, mean consultation rates increased from 22.3 to 49.3 per year for people with SMI and from 10.7 to 18.7 for controls. Focusing on specific consultation types, face-to-face consultations increased for people with SMI (from 8.8 in 2000/2001 to 10.9 in 2011/2012) but remained stable for control cases (4.8 in 2000/2001; 4.7 in 2011/2012). Across both groups, we observed a small increase in the mean number of phone consultations, from 0.6 to 1.2 for people with SMI and from 0.2 to 0.4 for control cases. The greatest increases within each group were observed for other consultations, from 12.8 to 37.3 for people with SMI and from 5.7 to 13.6 for control cases. The pattern of consultations rates did not differ greatly by type of SMI, but we generally observed higher rates across all consultation types for patients with bipolar and other affective disorders, compared with patients with schizophrenia (table 2). Females consulted more frequently than males in both groups, for all consultation types and in each year of the 12-year study period (see online appendix tables A3–4 and figure A1). As expected, consultation rates varied by age group. For patients aged 61 or over we observed face-to-face consultation rates of 13.0 and 7.1 in 2011/2012, for the SMI and control groups, respectively. For patients aged 41–60 and 18–40, the rates were 10.7 and 4.2 and 8.8 and 2.9, respectively (see online appendix tables A5–7).

**Table 2:** Mean (SD) consultation rates over time for all severe mental illness (SMI) cases, subgroups and control cases

	2000/2001	2001/2002	2002/2003	2003/2004	2004/2005	2005/2006	2006/2007	2007/2008	2008/2009	2009/2010	2010/2011	2011/2012
*All SMI cases*												
Face-to-face	8.8 (10.5)	8.8 (10.5)	8.7 (10.7)	9.2 (11.6)	9.4 (11.7)	9.5 (11.5)	9.8 (11.5)	9.2 (10.8)	9.5 (11.3)	9.8 (11.0)	10.0 (11.2)	10.9 (12.0)
Telephone	0.6 (2.6)	0.6 (2.4)	0.6 (2.5)	0.6 (2.5)	0.7 (2.6)	0.7 (2.8)	0.7 (2.4)	0.8 (2.3)	0.9 (2.6)	1.0 (2.8)	1.1 (2.9)	1.2 (3.3)
Other	12.8 (14.3)	15.3 (16.6)	18.1 (19.2)	20.9 (21.6)	25.1 (24.4)	27.2 (26.5)	29.8 (27.0)	30.6 (27.4)	32.3 (28.2)	33.4 (29.1)	34.5 (29.9)	37.3 (31.7)
Schizophrenia												
Face-to-face	8.3 (10.5)	8.4 (10.5)	8.1 (10.7)	8.5 (11.6)	8.7 (11.7)	8.7 (11.5)	9.0 (11.5)	8.2 (10.8)	8.4 (11.3)	8.8 (11.0)	9.2 (11.2)	10.0 (12.0)
Telephone	0.5 (2.6)	0.5 (2.4)	0.5 (2.5)	0.5 (2.5)	0.5 (2.6)	0.6 (2.8)	0.5 (2.4)	0.6 (2.3)	0.7 (2.6)	0.8 (2.8)	0.9 (2.9)	1.0 (3.3)
Other	11.8 (14.3)	14.0 (16.6)	16.4 (19.2)	19.2 (21.6)	23.5 (24.4)	25.6 (26.5)	28.0 (27.0)	29.0 (27.4)	30.6 (28.2)	32.1 (29.1)	33.5 (29.9)	36.5 (31.7)
Bipolar disorder												
Face-to-face	9.2 (10.5)	9.4 (10.5)	9.3 (10.7)	10.0 (11.6)	10.5 (11.7)	10.7 (11.5)	10.9 (11.5)	10.3 (10.8)	10.7 (11.3)	11.0 (11.0)	11.2 (11.2)	12.0 (12.0)
Telephone	0.6 (2.6)	0.7 (2.4)	0.7 (2.5)	0.7 (2.5)	0.8 (2.6)	0.9 (2.8)	0.9 (2.4)	0.9 (2.3)	1.0 (2.6)	1.1 (2.8)	1.2 (2.9)	1.3 (3.3)
Other	14.3 (14.3)	17.1 (16.6)	20.3 (19.2)	23.7 (21.6)	28.5 (24.4)	30.5 (26.5)	33.2 (27.0)	34.0 (27.4)	35.9 (28.2)	37.3 (29.1)	38.0 (29.9)	40.5 (31.7)
Other affective disorder												
Face-to-face	9.6 (10.5)	9.5 (10.5)	9.4 (10.7)	10.2 (11.6)	10.1 (11.7)	10.2 (11.5)	10.9 (11.5)	10.0 (10.8)	10.3 (11.3)	10.5 (11.0)	10.8 (11.2)	11.7 (12.0)
Telephone	0.5 (2.6)	0.6 (2.4)	0.7 (2.5)	0.7 (2.5)	0.8 (2.6)	0.9 (2.8)	0.9 (2.4)	0.9 (2.3)	1.0 (2.6)	1.1 (2.8)	1.3 (2.9)	1.5 (3.3)
Other	12.0 (14.3)	14.3 (16.6)	17.0 (19.2)	20.3 (21.6)	24.6 (24.4)	27.1 (26.5)	30.6 (27.0)	31.7 (27.4)	33.6 (28.2)	34.3 (29.1)	34.9 (29.9)	37.8 (31.7)
Other SMI type												
Face-to-face	8.5 (10.5)	8.4 (10.5)	8.4 (10.7)	8.6 (11.6)	8.8 (11.7)	8.8 (11.5)	9.0 (11.5)	8.6 (10.8)	8.9 (11.3)	9.1 (11.0)	9.3 (11.2)	10.2 (12.0)
Telephone	0.6 (2.6)	0.7 (2.4)	0.6 (2.5)	0.6 (2.5)	0.7 (2.6)	0.7 (2.8)	0.7 (2.4)	0.7 (2.3)	0.8 (2.6)	0.9 (2.8)	1.0 (2.9)	1.1 (3.3)
Other	12.4 (14.3)	15.0 (16.6)	17.6 (19.2)	20.1 (21.6)	23.5 (24.4)	25.4 (26.5)	27.9 (27.0)	28.4 (27.4)	29.9 (28.2)	30.7 (29.1)	32.2 (29.9)	34.9 (31.7)
*Control cases*												
Face-to-face	4.8 (6.6)	4.8 (6.7)	4.7 (6.7)	4.8 (6.9)	4.8 (7.2)	4.9 (7.3)	4.8 (7.2)	4.5 (6.9)	4.5 (6.8)	4.6 (6.9)	4.6 (7.0)	4.7 (7.4)
Telephone	0.2 (1.2)	0.3 (1.2)	0.3 (1.2)	0.3 (1.2)	0.3 (1.2)	0.3 (1.3)	0.3 (1.3)	0.3 (1.3)	0.3 (1.3)	0.4 (1.3)	0.4 (1.4)	0.4 (1.5)
Other	5.7 (9.2)	6.6 (10.3)	7.7 (12.0)	8.8 (13.6)	10.3 (15.4)	11.0 (16.6)	11.8 (17.2)	12.1 (17.5)	12.7 (18.3)	13.2 (18.9)	13.3 (19.3)	13.6 (19.8)

Regression analyses indicated a significant increase in overall consultation rates over time for both groups, after controlling for important covariates (see online appendix table A8). For people with SMI, the mean number of consultations was 92% higher in 2011/2012 compared with 2000/2001 (incidence rate ratio (IRR)=1.918; 95% CI 1.908 to 1.928). For control cases, the increase was smaller at 75% (IRR=1.746; 95% CI 1.738 to 1.753). Prior to the QOF introduction, the rate for controls was quite flat but was increasing for people with SMI; after QOF rates increased for both groups, but much more so for the SMI group (figure 1). Females tended to consult more frequently than men but the difference was smaller in the SMI group (IRR=1.236; 95% CI 1.222 to 1.251), than in the control group (IRR=1.540; 95% CI 1.532 to 1.547). All comorbidities were associated with more consultations but these effects were consistently smaller for the people with SMI. However, according to the third model, adjusted face-to-face consultation rates did not appear to increase over time for SMI or control patients, although adjusted other consultations did increase (table A2). Results from each of the three logistic regression models did not differ across the main and the two sensitivity analyses.

The results of the interrupted time-series analysis are presented in table 3. The pre-QOF trends in face-to-face and telephone consultations were fairly flat for both people with SMI and control patients (−0.04 (95% CI −0.1 to 0.01) and −0.05 (95% CI −0.08 to −0.01) per annum). However, we identified a mean step change of 0.56 in face-to-face consultations per person with SMI corresponding with the introduction of the QOF, although this was not statistically significant (95% CI −0.004 to 1.12), compared with a non-significant change of 0.16 (95% CI −0.045 to 0.36) for controls. We also observed a statistically significant increase from pre-QOF to post-QOF in the annual rate of face-to-face consultations for people with SMI, of 0.19 consultations (95% CI 0.02 to 0.36) per patient per year, compared with 0.01 (95% CI −0.04 to 0.07) for controls (coefficients across models statistically different, p=0.022). For telephone consultations, step changes were not statistically significant for either group, but we observed small but significant annual trend increases 0.06 (95% CI 0.02 to 0.09) for people with SMI and 0.01 (95% CI 0.005 to 0.02) for controls (coefficients across models statistically different, p=0.004).

**Table 3:** Interrupted time-series analyses results, reporting changes in average number (95% CI) of consultations per patient*

	SMI	Controls	p Value†
Face-to-face			
Starting level	8.88 (8.75 to 9.00)	4.87 (4.79 to 4.95)	<0.001
Pre-QOF annual trend	−0.04 (−0.10 to 0.01)	−0.05 (−0.08 to −0.01)	0.832
Step change	0.56 (−0.004 to 1.12)	0.16 (−0.04 to 0.36)	0.113
Telephone			
Post-QOF annual trend change†	0.19 (0.02 to 0.36)	0.01 (−0.04 to 0.07)	0.022
Starting level	0.59 (0.53 to 0.64)	0.23 (0.22 to 0.25)	<0.001
Pre-QOF annual trend	0.01 (−0.01 to 0.04)	0.01 (0.001 to 0.01)	0.639
Step change	−0.02 (−0.16 to 0.12)	0.0002 (−0.03 to 0.03)	0.719
Post-QOF annual trend change†	0.06 (0.02 to 0.09)	0.01 (0.005 to 0.02)	0.004
Other			
Starting level	10.20 (10.02 to 10.38)	4.66 (4.52 to 4.79)	<0.001
Pre-QOF annual trend	2.61 (2.53 to 2.69)	1.01 (0.95 to 1.07)	<0.001
Step change	2.39 (1.34 to 3.44)	0.95 (0.45 to 1.45)	0.003
Post-QOF annual trend change‡	−1.00 (−1.25 to −0.75)	−0.55 (−0.68 to −0.42)	<0.001

*2003/2004 was excluded from analyses since it was considered a preparatory year.

†Comparing coefficients across models using the z-value formula 
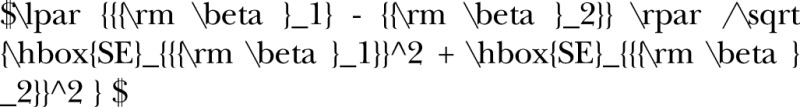
.

‡Change compared with pre-QOF trend.

SMI, severe mental illness; QOF, Quality and Outcomes Framework.

The pre-QOF trend for other types of consultations was increasing, at 2.61 (95% CI 2.53 to 2.69) per annum for people with SMI and 1.01 (95% CI 0.95 to 1.07) for controls (coefficients across models statistically different, p<0.001). The step change when QOF was introduced was also significant for both groups, 2.39 (95% CI 1.34 to 3.44) for people with SMI and 0.95 (95% CI 0.45 to 1.45) for controls, and the coefficients were statistically different (p=0.003). Post-QOF, the annual rate of increase significantly declined for both groups, −1.00 (95% CI −1.25 to −0.75) for people with SMI and −0.55 (95% CI −0.68 to −0.42) for controls, though in absolute terms numbers of other types of consultation continued to increase (coefficients across models statistically different, p<0.001). There was no evidence that any pre-QOF and post-QOF trend deviated from linearity. Result patterns across the main and two sensitivity analyses were consistent.

## Discussion

We found that people with SMI consulted in primary care more often than people without across the 12-year study period. The differences in consultation rates increased following the introduction of the QOF in 2004. Consultation rates for people with SMI increased across all three consultation types (face-to-face, telephone and other), and were consistently higher than what we observed for control cases, with the biggest increases in other consultations. When controlling for the preintervention trend through the interrupted time-series analysis, we observed increases in face-to-face consultations for people with SMI which were not observed for the controls, while increases in telephone consultations appeared larger in the SMI group. All other types of consultations, including administrative tasks, were on the increase before the introduction of the QOF for both groups and more so for people with SMI, possibly reflecting the increasing computerisation of primary care. Nevertheless we still observed a step increase with the introduction of the QOF, larger for people with SMI, although the rates of increase slowed down over time for both groups. These findings are in agreement with the hypothesis that the introduction of the QOF successfully incentivised GPs to monitor the health of people with SMI more frequently. Over time, we also observed a great increase in the average number of reported comorbidities for the SMI group. However, this could be attributable to improved case finding driven by the increase in face-to-face consultations or due to improved recording by practices, rather than any actual general deterioration in the health of people with SMI. The increase in reported comorbidities also explains the low adjusted consultation rates post-QOF, since people with SMI appeared less healthy post-QOF.

### Strengths and limitations

This is the largest longitudinal study of consultation rate patterns for people with SMI in UK primary care. However, there are important limitations. First, this is an observational study and we cannot be certain that the increases we observed can be fully attributed to the introduction of the QOF incentivisation scheme. They might have emerged regardless, as a result of increased awareness of the unmet health needs of people with SMI, or later initiatives.^34^ Second, we cannot rule out that the increase in comorbidities for the SMI group does not indicate a real deterioration in health, rather than better case finding. However, the fact that other characteristics of the population (especially age) remain unchanged over time makes the real health deterioration scenario unlikely. For this reason, we principally focus on unadjusted analyses, since we feel that the health deterioration assumption on which analyses controlled for comorbidities are based is much less likely. Third, any study of this nature is limited by the reliability and accuracy of the data in the patient's electronic record. We are confident about the reliability of the recorded patient contact data and patient characteristics. However, less is known about the accuracy of the information on type of consultation, and primary care staff may be inclined to record face-to-face as the default option. If so, our data will have overestimated face-to-face consultations. Although consultation patterns may differ across health professions, we did not use consulting professional data due to reliability uncertainty. Fourth, we could not assess the content of consultations, and so do not know whether they adequately address the needs of patients. Fifth, for practical reasons and completeness, we aggregated various consultations types into an ‘other’ category. Although this contains vastly heterogeneous consultation types, which we did not aim to investigate separately, as well as purely administrative events, it does provide some insight into the increased overall workload. Finally, the CPRD collects data from practices using the Vision clinical system and recording activity may differ for practices using other systems,^35^ although we would not expect that potential variation to affect our findings.

### Findings

Women and older people were more likely to consult, in agreement with previous research.^18^ ^29^ Analysis of the QResearch database reported an annual face-to-face consultation rate of 5.4 for the general population in 2008,^18^ higher than our 2008/2009 estimate of 4.6. This discrepancy may be explained by differences in local deprivation and clinical computer systems, as well as by the different characteristics in our matched control group and the general population. A previous smaller study reported an average face-to-face consultation rate for people with SMI with GPs and nurses of 6.7, for 2008/2009.^2^ Although our face-to-face consultation rate is higher at 9.5 for the same year, we included consultations with all primary healthcare professionals.

People with SMI are more likely to die prematurely^13^ ^36^ and to have comorbid health conditions.^11^ ^37^ Primary care has a pivotal role to play in the care of people with SMI,^2^ especially in terms of physical health monitoring and providing preventative services.^38^ ^39^ Nevertheless, it is worth highlighting that within a year of first diagnosis, diagnosis is not finalised for one in five patients. It is also unsurprising that consultation rates are higher if need is translated into greater service utilisation. What is unclear is whether primary care consultations are adequately addressing this need. However, our findings suggest that people with SMI generally have the opportunity to be in receipt of the checks required by QOF, given the observed face-to-face consultation rates, although the effectiveness of these checks in improving patient health is still being debated.^40–43^ In line with the national well-being and recovery agenda, consultation rates may continue to increase further as Community Mental Health Teams are encouraged to discharge all but the most severe cases back to primary care. Conversely, the removal of cardiometabolic QOF indicators (requiring annual recording of weight, blood cholesterol and glucose) in 2014 might curb such an increase. Recent evidence suggests that removal of QOF incentives has had negligible effects on recorded quality of care, both short-term and medium-term.^22^ However, most previously removed QOF activities were still indirectly incentivised and further research is required to assess the long-term impact of removing SMI indicators.

## Conclusions

Consultation rates for people with SMI increased over time, for all three consultation types. The increase was greater after the introduction of financial incentives in 2004. It seems reasonable to conclude that the introduction of the scheme incentivised practices to assess people with SMI more often, and that this led to better case findings for comorbidities.
